# Causes of Dental Deformities, with Other Incidental Phenomena, Depending on Abnormal States of He Formative Process during Dentition

**Published:** 1855-10

**Authors:** J. L. Levison

**Affiliations:** Brighton.


### ARTICLE II.

*Causes of Dental Deformities, with other incidental Phenomena, depending on Abnormal States of he Formative Process during Dentition.* By J. L. LEVISON, D. D. S., &c. &c., Brighton.

[Continued from page 230.*]

THERE are other cases where the bodily powers are implicated, besides those which result from the too early training of the mental faculties. These cases are to be found among the offspring of drunkards and debauchees, and who indicate their diseased inheritance, by rickets, scrofula, diseased spine, fetid mouths, and imperfectly formed teeth, which, with all the bones of the body are deficient of calcareous salts, essential to give them durability. And as these are the signs of a want of stamina in the system, many of the morbid symptoms are aggravated, and the teeth soon become brittle and carious. But if under the body-destroying tendencies intimated, there should happen to be nice looking teeth, with good shape and pearly color they will often, under such circumstances, become rapidly destroyed; forming no exception to the poet's lament,

> "All that's bright must fade,
> The brightest, still the fleetest."

But to proceed: it is imperative in practice to learn how to distinguish the predisposing causes of a disease in which there are so many phases in common.

For instance, many of the symptoms which were manifested in cases of the neglect of the physical laws, mentioned in the previous communication, are found in cases of *mollites ossium.* In this latter form of disease, often to be found in the offspring of sensualists, the teeth suffer decay from an absolute deficiency of osseous matter, and they are in consequence soon doomed to the destructive process of caries. One case I shall cite, as it furnishes important *data*, and practically discriminates the actual resemblance or difference in the general and special phenomena, and enables a correct *diagnosis* to be formed. A sickly, rickety young lady was brought to me for my advice, in consequence of there not being more than ten or eleven teeth in both jaws. These teeth had but a thin coating of enamel, and the dentine was glassy in appearance from a deficiency of earthy matter. The young lady was sickly and palid—her jaws were much contracted and very thin, the muscles of the whole body were flabby, the legs were bowed, the abdomen large, owing to the abnormal enlargement of the mysenteric glands, giving an aged aspect to this diminutive person. The pulse was irregular and very tardy, indicating by this outward and visible sign a state of imperfect circulation. The progenitor was an elderly man, very sickly, scrofulous, and attenuated. He had evidently been a victim in early life to some abuse of the functions of the cerebellum.

Can this sad picture of imperfect physical power, and its attendant defective bodily development, be contemplated without pain. Particularly when contrasted with a graceful, active, energetic condition of a child born of healthy parents. The early life of such an one is a series of physical gratification; the merry laugh, and the almost incessant motions of its well formed limbs, its natural appetite, and rapid digestion, had made its life one of truly happy childhood.

But the poor sickly being spoken of, having inherited a diseased condition of body, moves listlessly and with an unsteady gait, a child full of premature care and precocious suffering.

This sketch is not overdrawn. It is a faithful copy of the effects of parental abuse, being visited on the unfortunate offspring. And in practice a case like this should be distinguished from those given in my preceding paper.

But in all instances of defective stamina, it seems impossible to render existence felicitous. From whatever source may be the cause which enfeeble the bodily powers, there is an incapacity to feel the pure and exhilarating pleasure of active physical enjoyment. There is besides not sufficient moral energy; sunshine and shade are alike unheeded—the warmth of the first is too debilitating, and the gloom of the latter too chilling. Persons who from infancy are thus diseased, will look on the beauties of Flora without any emotion of delight, whilst the nobler forms and graceful motions of some of the Fauna, excite the reverse of pleasurable sensations from their own incapacity to bound like the children of vigorous health. When, therefore, we contemplate such victims, immolated to disease, we are furnished with indubitable evidence, "that the sins of the fathers (moral and physical) are visited on the children." Hence we labor under a species of moral obligation to warn and instruct society, that men may restrain their sensuality, and not propagate beings who, from the moment they breathe the "air of heaven," suffer the penalty for the selfishness of their progenitors, and from birth to death drag out a miserable and unfelicitous existence. Alas! for poor human nature.

Among the many advantages which must result from the preceding views, there is one which should be mentioned in a journal devoted to dental surgery and pathology, namely, in cases of chronic tooth-ache, arising from the unnatural pressure of crowded teeth, so closely compacted within the arches of the *alveoli* in the jaws, so as to induce a constant inflammatory action. Sometimes, in strumous persons there may be caused a spongy state of the gums, the recession of the latter, and the exposure of the dentine, (unprotected with enamel,) and allowing the neutral salivary salts and foreign matter to be deposited; which, by increasing the irritation, ultimately excite the absorbents, and there may be a removal of the sockets, or caries may be induced at the necks of each tooth.

But there is another affection so invidious, and yet such a source of suffering, that this paper would be defective if it were omitted.

Hence, we shall describe a case or two as furnishing indubitable proof that the contracted jaw is not only a cause of deformity, but also very often a source of disturbance to the general health.

Some years since, during my residence at Doncaster, in Yorkshire, a young lady consulted me, having suffered for three or four years constant tooth-ache, which was more or less intense and aggravated by moral causes, on any slight derangement of the stomach. She said, "I have had the advice of physicians, surgeons, and dentists, and without any benefit, nay I begin to despair of any mitigation." She was about twenty-five or six years old, a nervo-sanguineous temperament, with a well-formed chest and brain, and the appearance of a good natural constitution, and her intellectual and moral attributes had been cultivated, so that besides being highly intelligent, she was urbane, amiable, and practically benevolent.

When she called, she appeared much excited, her face was flushed, and her eyes more brilliant than they would be in her ordinary state; evidently arising, however, from cerebral irritation, but whether from any local cause, or from reflex nervous action, or from the vigilance and restlessness induced by her chronic tooth-ache, were in the first instance matter of speculation. As is my wont in obscure cases, I subjected my patient to a rigid cross-examination, inspected afterwards her mouth, and then formed my *diagnosis*, and told the patient I had no doubt that I could soon give her relief. To which she replied, probably more candidly than courteously, "this I do not anticipate, although I came to you under a forlorn hope that you could mitigate my sufferings." Before proceeding with the simple treatment and immediate success, it is well to state, it was evident that great care had been bestowed during the second dentition, the anterior arches were well formed, and the teeth beautifully arranged in their natural order, but so closely packed together, that it was impossible to pass between them the thinnest thread or silk; and at the posterior ends of the lower jaw the *dentes sapientiæ* were forced under the interior ridge of the coronoid process; and on the upper the same teeth were forced out of the curve, being pressed outwards towards the cheeks.

I proposed extracting a tooth from each jaw, but, though a sensible woman, she objected, from a notion that it would be removing the key-stone of the arch; and that some marked alteration would take place to mar the symmetry of the teeth, and as a consequence the expression of her mouth.

Now as she was a very handsome personage, this line of argument seemed most natural, and formed a sufficient reason for a negative! Yet as my own conviction was, *a priori*, that all her suffering arose from dental pressure, I next suggested that she should permit me to separate her teeth by means of a dividing file—to this she put in a demurrer, to which I replied, proving that very little of the enamel would be removed, (as the file was not thicker than writing paper,) and the operation might be done with perfeet impunity. This argument, after due consideration, was admitted to be valid, and the lady assented.

I then divided every two teeth, making just enough space so as to admit a piece of thread to pass without resistance or obstruction.

Never shall I forget either her expression or her gratitude. As soon as my operation was over, she said, "O sir! I have lost the painful symptoms! I have not felt so easy for years!" So great was her joy, that tears filled her eyes. I saw her repeatedly afterwards, and she had not any return of tooth-ache. Her features, which were of a classic form, assumed their natural sweet expression, her health greatly improved, and she could continue, without any sensation of lassitude, her passion for literature, and this simply because her sleep was sound and the cerebral functions normal.

I am tempted to give one more from my note-book, as offering some matter for reflection and comment.

Mr. P——, a fine, tall, athletic young man, had been for two or three years a soldier and bought off by his relatives, subsequently became a teacher of gymnastics in Brighton. He was above six feet two inches in height, with an enormous chest and upper extremities, and whose muscular system presented one of the most perfectly developed models of strength, from their dense and iron-hardness, yet he suffered from tooth-ache, and came with a dolorous face to ask me to stop him some teeth.

On looking into his capacious mouth,* I perceived two and thirty teeth, without a speck or blemish—the jaws were ample, the teeth arranged in curves, and the palatal bones finely arched. I told him he had not a tooth that required stopping. He said that this must be an error, as he had suffered tooth-ache more or less for more than two years. It appeared that this, like the preceding case, arose from the close proximity of the teeth, which were jammed together, hence the chronic tooth-ache he had endured. In Mr. P——'s case, I did not propose extracting any teeth, but I separated a half dozen in each jaw, and had the satisfaction to have my patient declare that he was free from pain, as if by magic! He sat for a few minutes champing his enormous jaws together, and with a merry voice he made the following statement, "I seem as if released from some strong controlling influence; for I can now move my jaws easily and without pain. But for a very long time my sensations have been as if I wanted to grind the teeth together, to get rid of the irritation, and my gums were always hot and itching, which made me desire to rub something hard against them. But now I'm all right!" Probably the most scientific exposition could not render more graphic the inconvenience and annoyance which he endured from the undue pressure on the dental arches. This condition had interfered with his comfort and affected his spirits. And it may be added, that the chronic inflammatory action, though it did not induce caries in his large and filbert-shaped teeth, yet disease would soon have been developed, judging from their color, but which tendency succumbed to the operation, as the teeth soon became white, and brilliantly pol-

*I took models of Mr. P——'s upper and under jaws, and will send them, with the casts figured in my last communication, for the museum of the Baltimore College, when there is any chance of their going safely.—J. L. L.

ished, as there was removed that under pressure which had interfered with their healthy and normal functions, and he often showed me, with some exultation, that they were not afterwards affected by either local or remote disturbing causes.

As this communication will occupy a large space in your valuable Journal, I reserve some other facts connected with dental pressure for some other opportunity.

[To be continued.]

